# Temporal serum metabolomic and lipidomic analyses distinguish patients with access-related hand disability following arteriovenous fistula creation

**DOI:** 10.1038/s41598-023-43664-z

**Published:** 2023-10-05

**Authors:** Ram B. Khattri, Lauryn Z. Louis, Kyoungrae Kim, Erik M. Anderson, Brian Fazzone, Kenneth C. Harland, Qiongyao Hu, Kerri A. O’Malley, Scott A. Berceli, James Wymer, Terence E. Ryan, Salvatore T. Scali

**Affiliations:** 1 Department of Applied Physiology and Kinesiology, University of Florida, Gainesville, FL 32611 USA; 2 Division of Vascular Surgery and Endovascular Therapy, University of Florida, Gainesville, FL 32611 USA; 3 Center for Exercise Science, University of Florida, Gainesville, FL 32611 USA; 4 Department of Neurology, University of Florida, Gainesville, FL 32611 USA; 5 Malcom Randall Veteran Affairs Medical Center, Gainesville, FL USA; 6 Gainesville, USA

**Keywords:** Metabolomics, Blood flow, Chronic kidney disease, Haemodialysis, Translational research

## Abstract

For end-stage kidney disease (ESKD) patients, hemodialysis requires durable vascular access which is often surgically created using an arteriovenous fistula (AVF). However, some ESKD patients that undergo AVF placement develop access-related hand dysfunction (ARHD) through unknown mechanisms. In this study, we sought to determine if changes in the serum metabolome could distinguish ESKD patients that develop ARHD from those that have normal hand function following AVF creation. Forty-five ESKD patients that underwent first-time AVF creation were included in this study. Blood samples were obtained pre-operatively and 6-weeks post-operatively and metabolites were extracted and analyzed using nuclear magnetic resonance spectroscopy. Patients underwent thorough examination of hand function at both timepoints using the following assessments: grip strength manometry, dexterity, sensation, motor and sensory nerve conduction testing, hemodynamics, and the Disabilities of the Arm, Shoulder, and Hand (DASH) questionnaire. Nineteen of the forty-five patients displayed overt weakness using grip strength manometry (*P* < 0.0001). Unfortunately, the serum metabolome was indistinguishable between patients with and without weakness following AVF surgery. However, a significant correlation was found between the change in tryptophan levels and the change in grip strength suggesting a possible role of tryptophan-derived uremic metabolites in post-AVF hand-associated weakness. Compared to grip strength, changes in dexterity and sensation were smaller than those observed in grip strength, however, post-operative decreases in phenylalanine, glycine, and alanine were unique to patients that developed signs of motor or sensory disability following AVF creation.

## Introduction

A reliable dialysis access is a lifeline for patients with end-stage kidney disease (ESKD). Notably, hemodialysis is the most prevalent form of renal replacement therapy and recently updated clinical practice guidelines still generally recommend surgical creation of an arteriovenous fistula (AVF), especially in younger patients with good life-expectancy. While AVF placement is critical for establishing durable hemoaccess, a significant proportion of patients develop hand disability ipsilateral to the newly created dialysis-access that includes a spectrum of symptoms ranging from sensory impairment, grip weakness, motor discoordination to even more severe presentations of rest pain or gangrene^1^, which collectively is often termed access-related hand ischemia (ARHI), or ‘steal syndrome’. In the most severe presentations, ARHI mandates surgical intervention; however, residual hand symptoms in up to 20% of remediated patients highlights the lack of fundamental mechanistic understanding of what biologic drivers are responsible for the significant variation in the vulnerability and manifestations of the observed clinical phenotype^1–3^. Moreover, recent temporal phenotyping and multivariable analysis of hand function following AVF placement reveal that hemodynamic changes alone do not adequately account for the spectrum of access-related hand dysfunction (ARHD) that is commonly detected among these patients^4^. The lack of a predictable association between AVF-induced hemodynamic perturbations and ARHD further contributes to the inability to accurately identify high-risk patients which has a significant negative impact on quality of life.

A defining metabolic feature of ESKD is presence of a toxic systemic milieu driven by uremic metabolite accumulation, metabolic acidosis, and inflammation^5–12^. However, whether these factors contribute to the development of ARHD is unknown. The emergence of metabolomics has increased our understanding of how cells/tissues use metabolites to both support cell function and as signals for communication. To this end, the objective of this study was to determine if analysis of the serum metabolome prior to- and after AVF surgery could identify metabolic alterations that distinguish patients with and without ARHD. To accomplish this, we analyzed the metabolomic profile of a well-characterized group of ESKD patients that underwent surgical creation of an AVF^4^ using nuclear magnetic resonance (NMR) spectroscopy.

## Methods

### Study population

A detailed description of the patient population examined in this study is provided in our previous report^4^. Briefly, serum was obtained from a total of 45 patients with ESKD that underwent first-time AVF creation at the University of Florida. Physical and clinical characteristics of the patients is shown in Table 1. Serum was obtained using standard venipuncture pre-operatively and 6 weeks post-operatively, allowed to clot, and centrifuged before cryo-storage of the resulting supernatant. Patients underwent an array of tests at the same time points evaluating hand function including grip strength manometry, dexterity (Purdue Pegboard), sensation (Semmer-Weinstein Monofilament testing), motor and sensory nerve conduction testing, hemodynamics (wrist and digit pressures), as well as the Disabilities of the Arm, Shoulder, and Hand (DASH) questionnaire, as previously described^4^. This study was approved by the Institutional review board (IRB) at the University of Florida reviewed and informed consent was obtained from all patients (IRB#140-2013, IRB#556-2009). This study was performed in accordance with the Declaration of Helinksi.

**Table 1:** Physical and clinical characteristics of ESKD patients.

Physical characteristics—no. (%)	
Sample size	45
Age—years (mean ± SD)	59.6 ± 13.5
Female sex	9 (20)
Body mass index—kg/m^2^ (mean ± SD)	30.06 ± 7.13
Race—no. (%)	
Black	17 (38)
White	27 (60)
Other	1 (2)
Clinical characteristic – mean ± SD	
eGFR – (mL/min/1.73 m^2^) prior to AVF	13.24 ± 6.09
Etiology of kidney disease—no. (%)	
Hypertensive nephropathy	7 (16)
Diabetic nephropathy	15 (33)
Glomerulonephritis	3 (7)
Idiopathic	16 (36)
Other	4 (8)
Co-morbid conditions—no. (%)	
Hypertension	44 (98)
Diabetes	30 (67)
Hyperlipidemia	27 (60)
Former/active smoking	27 (60)
AVF configuration—no. (%)	
Brachiocephalic	19 (43)
Radiocephalic	14 (32)
Brachiobasilic—1-stage	8 (17)
Brachiobasilic—2-stage	4 (8)
Duration on renal replacement therapy at the time of surgery—no. (%)	
Not on renal replacement therapy	24 (53)
< 6 months	14 (31)
> 6 months	7 (16)

### Metabolite extraction

To remove serum proteins (i.e., albumin) that broaden NMR peaks and complicate metabolite quantification, we initially employed a modified FOLCH extraction to separate water-soluble and lipid-soluble metabolites from 200 μl of serum as previously described^13,14^. In short, 200 µl of serum was mixed with 3 ml of ice cold chloroform:methanol mixture (2:1 v/v) in a 20 ml glass vial. The mixture was vortexed for 2 min and placed into an ice bath for 15 min to allow separation of the aqueous phase and lipid phases. Next, 1 ml of ice-cold ultrapure water was added and sample was vortexed again for 3 min and subsequently placed into an ice bath for another 45 min to allow complete phase separation. After the phase separation, the upper aqueous portion (water/methanol layer) was transferred to a new falcon tube. To the lower lipid phase (chloroform layer), 1 ml of ice-cold ultrapure water was added and the samples was vortexed for 3 min and then placed into the ice bath for 45 min. The aqueous phase (water/methanol layer) from this second separation was combined with the previous aqueous collection following the first phase separation and was dried overnight using Labconco freeze drier (Labconco Corporation, MO, USA). The final remaining lipid (organic) phase was dried using inert nitrogen gas. The dried aqueous phase was subjected to a second acetonitrile:isopropanol:water (3:3:2 vol/vol/vol) extraction done by resuspending the dried samples in 1 ml of ice cold acetonitrile:isopropanol:water (3:3:2 vol/vol/vol) followed by vortexed for 2 min. The mixture was transferred to 1.5 ml epi-tube and centrifuged for 30 min (13,000 rpm, 4 °C). The supernatant was transferred to a new vial and lyophilized overnight (Labconco Corporation, Kansas, MO, USA). The lyophilized aqueous phase powder was dissolved a second time in acetonitrile:water (1:1 vol/vol), vortexed for 2 min, centrifuged (13,000 rpm, 4 °C, 30 min), and the supernatant was lyophilized for a final time. The resulting aqueous phase dry powder along with the lipid phase dry powder were stored at − 80 °C until analysis.

### NMR-based metabolomics

The lyophilized powders of aqueous phase samples were dissolved in 500 µL of 50 mM of phosphate buffer (pH of 7.4) in a 100% deuterated environment supplemented with 0.5 mM D6-4,4-dimethyl-4-silapentane-1-sulfonic acid (D6-DSS), 2 mM ethylene diamine tetraacetic acid (EDTA), and 0.02% sodium azide. The dry powders of lipid phase samples were dissolved in 600 µL of deuterated chloroform (CDCl_3_) containing 10 mM of pyrazine as an internal standard (chemical shift = 8.61 ppm). All samples were loaded into 5 mm outer diameter (O.D.) NMR tubes. A 600 MHz Bruker Avance III system (Bruker BioSpin Corporation, Billerica, MA, USA) with a 5-mm TCl CryoProbe was used to acquire all 1-dimensional (1D) and 2-dimensional (2D) NMR spectra. 1D ^1^H spectra were acquired with the 1D nuclear Overhauser effect spectroscopy (NOESY) pulse sequence (noesypr1d)^15^, utilizing identical parameters described previously^13,14,16^. Two dimensional spectra including heteronuclear single quantum coherence (HSQC), heteronuclear multiple bond coherence (HMBC), correlated spectroscopy (COSY), and total correlated spectroscopy (TOCSY) were acquired using the standard Bruker library, as well as previous work^17^ to confirm metabolite identity. All spectra were acquired at room temperature (25 ± 0.1 °C). Detailed methods involved in NMR acquisition and processing have been previously reported^13,18^.

### NMR data processing and analysis

MestReNova (14.1.2-25,024) software (Mestrelab Research, S.L., Santago de Compostela, Spain) was used to process all NMR spectra. For 1D NOESY spectra, line broadening of 0.22 Hz and 64 k data points zero filling were applied before Fourier transformation. Before extracting peak integral areas for quantitative purposes, 1D spectra were phase and baseline (Spline method) corrected, referenced, and normalized. All aqueous phase spectra were referenced and normalized with the D6-DSS internal NMR reference at 0.00 ppm. For lipid phase spectra, the CDCl_3_ peak at 7.26 ppm was used to reference all peaks and the pyrazine peak (at 8.61 ppm) was used for normalization. For peaks with overlap, Chenomx Suite 8.6 NMR software (Chenomx, Inc., Edmonton, AB, Canada) was used to determine concentrations.

### Statistical analysis

Principal component analysis (PCA) was performed using the web-based platform Metaboanalyst 5.0 (https://www.metaboanalyst.ca/). Within Metaboanalyst 5.0, NMR noise was excluded from false discovery rate (FDR) corrected data by applying Interquartile range (IGR) filtering. Comparisons between two groups were performed using unpaired Student’s* t*-test with FDR correction for multiple comparisons. Metabolite changes between groups were presented as a fold-change of the group without ARHD and heatmaps were generated using GraphPad Prism (version 9.2.0 (332), GraphPad Software, San Diego, CA, USA). In all cases, a *P* < 0.05 was considered statistically significant.

## Results

**Figure 1:**
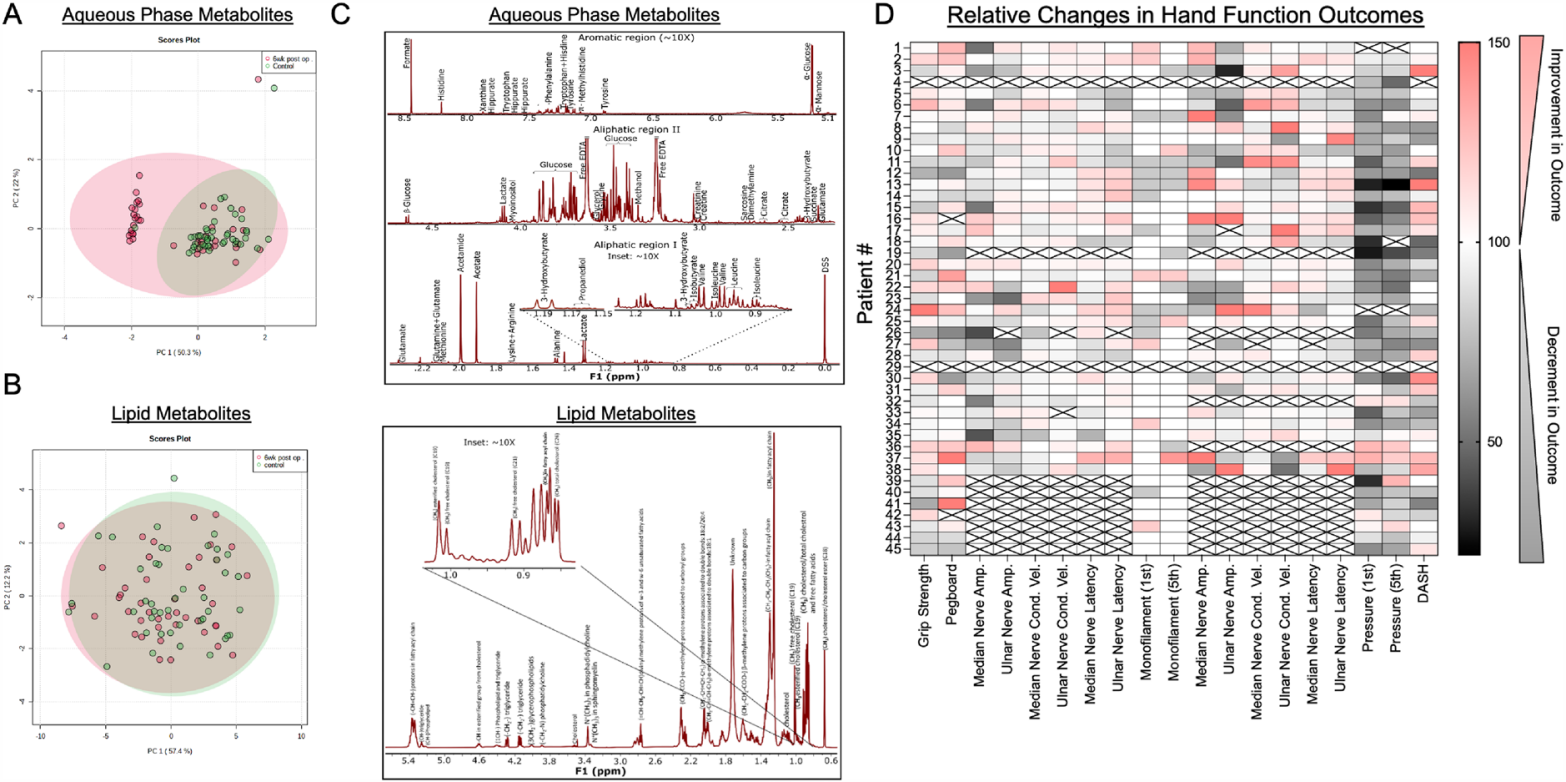
Metabolomic and Lipidomic Changes in Relation to Access Related Hand Disability. (**A**) Principal component analysis of aqueous phase metabolites obtained from blood samples collected pre-operatively (Control) and 6 weeks post-AVF surgery. (**B**) Principal component analysis of organic phase (lipid) metabolites obtained from blood samples collected pre-operatively (Control) and 6 weeks post-AVF surgery. (**C**) Representative annotated NMR spectra for aqueous and lipid metabolites extractions. (**D**) A heatmap showing the relative percent change (post-operative relative to pre-operative) across outcomes measured to assess hand disability for each individual patient.

Using ^1^H NMR to quantify metabolites, 38 aqueous phase and 18 lipid phase metabolites were reliably identified in all serum samples. We subjected metabolite concentrations at baseline and 6 weeks post-operatively to principal components analysis (PCA) using FDR corrected data to reduce dimensionality and examine variance between the time points and patients. PCA analysis showed overlapping clustering for aqueous phase metabolites for the majority of patients, although a clearly distinguishable group was observed in the post-operative samples (Fig. 1A). In contrast, lipid phase metabolites displayed overlapping clusters indicating that baseline and post-operative lipid metabolites were similar (Fig. 1B). Representative annotated NMR spectra with metabolites identified for both aqueous and lipid (organic) phase extracts are shown in Fig. 1C. Additionally, relative percent changes (post-operative vs. pre-operative) in phenotypic outcomes assessing hand function are shown in Fig. 1D for each individual patient within this study.

### The serum metabolome in ARHD patients with grip strength weakness is indistinguishable from those without ARHD

**Figure 2:**
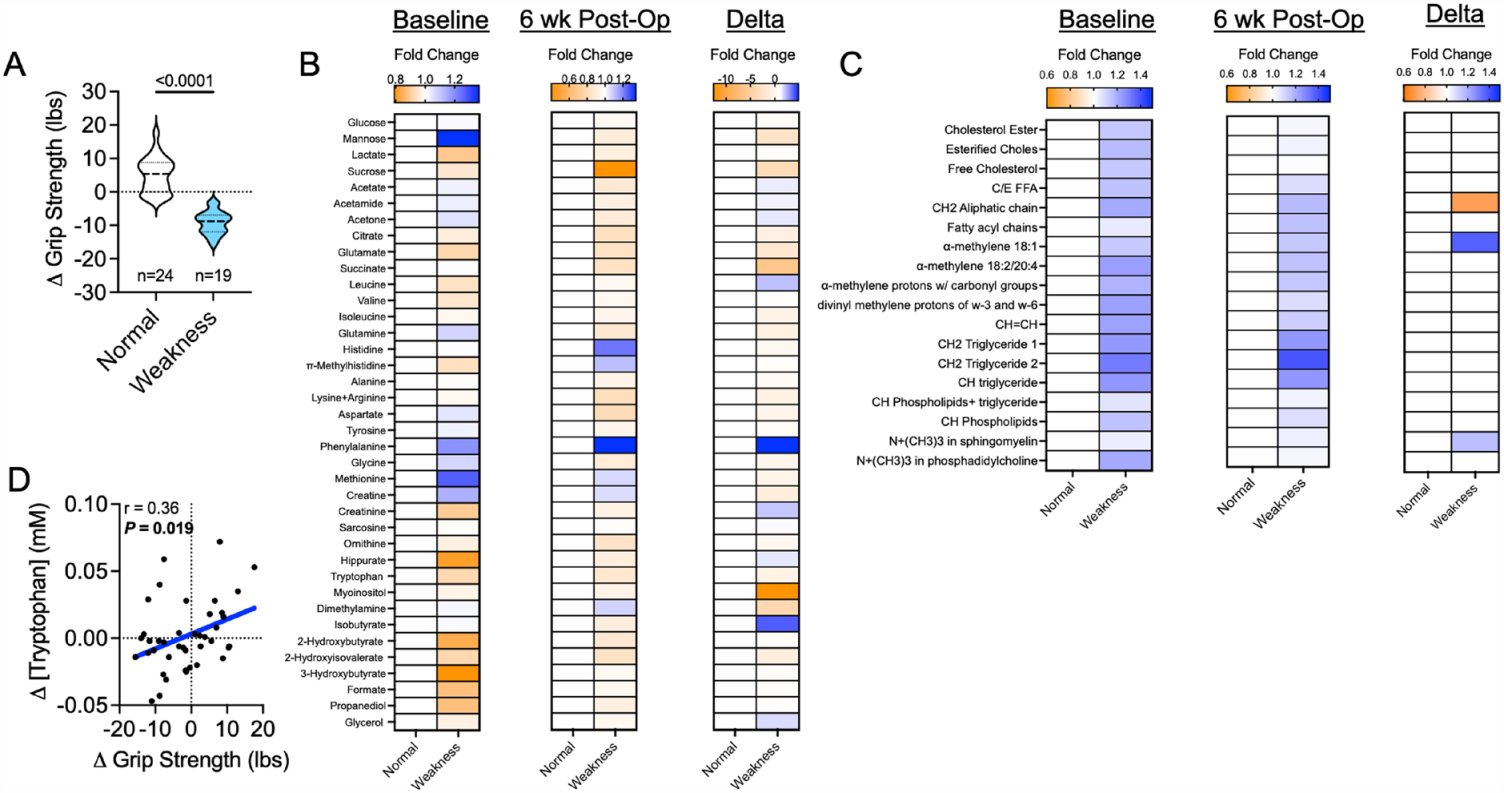
The serum metabolome is indistinguishable between patients with and without weakness following AVF surgery. (**A**) Violin plots shown the change in grip strength (post-operative minus pre-operative) for patients classified as ‘normal’ and those with ‘weakness’. (**B**) Heatmaps showing the fold change in aqueous phase metabolites at baseline (pre-operative), 6 weeks post-operatively, and the change for patients classified as ‘normal’ and those with ‘weakness’. (**C**) Heatmaps showing the fold change in lipid phase metabolites at baseline (pre-operative), 6 weeks post-operatively, and the change for patients classified as ‘normal’ and those with ‘weakness’. (**D**) Correlation between the change in serum tryptophan levels and the change in grip strength. Comparisons between two groups were performed using an unpaired Student’s* t*-test. Metabolite comparisons were performed using an unpaired Student’s* t*-test. with FDR correction for multiple comparisons.

Next, we assigned patients to either ‘normal’ or ‘weakness’ groups based on changes in grip strength following AVF creation. Accordingly, 19 patients displayed a decline in grip strength whereas 24 patients exhibited either no change or a slight increase in grip strength between the preoperative and 6-week postoperative time points. Two patients did not have baseline and post-operative grip strength measures and were exclude from subsequent downstream analyses. Expectedly, patients that developed post-operative hand weakness had a significant difference in their delta grip strength compared to those without weakness (*P* < 0.0001, Fig. 2A). Next, we expressed the aqueous phase metabolites in the ARHD patients with weakness as a fold change from the normal group at baseline, 6 weeks post-operatively, and the delta (post-operative minus baseline). Statistical comparisons of metabolite changes between these groups revealed that no significant differences were observed, indicating that the serum metabolome in ARHD patients with weakness is indistinguishable from those without ARHD/weakness (Fig. 2B). Similarly, the serum lipid metabolites measured were also not different between ARHD patients with weakness and those without ARHD (Fig. 2C). Pearson correlational analyses were then performed to determine if any metabolites were associated with grip strength across the entire patient cohort. Results of this analysis identified a significant correlation between the change in grip strength and change in tryptophan concentrations, demonstrating that among patients who developed weakness, they also had a decline in circulating tryptophan post-operatively (Fig. 2D).

### Several metabolites distinguish ARHD patients with mild motor discoordination from those without dexterity/motor impairment

**Figure 3:**
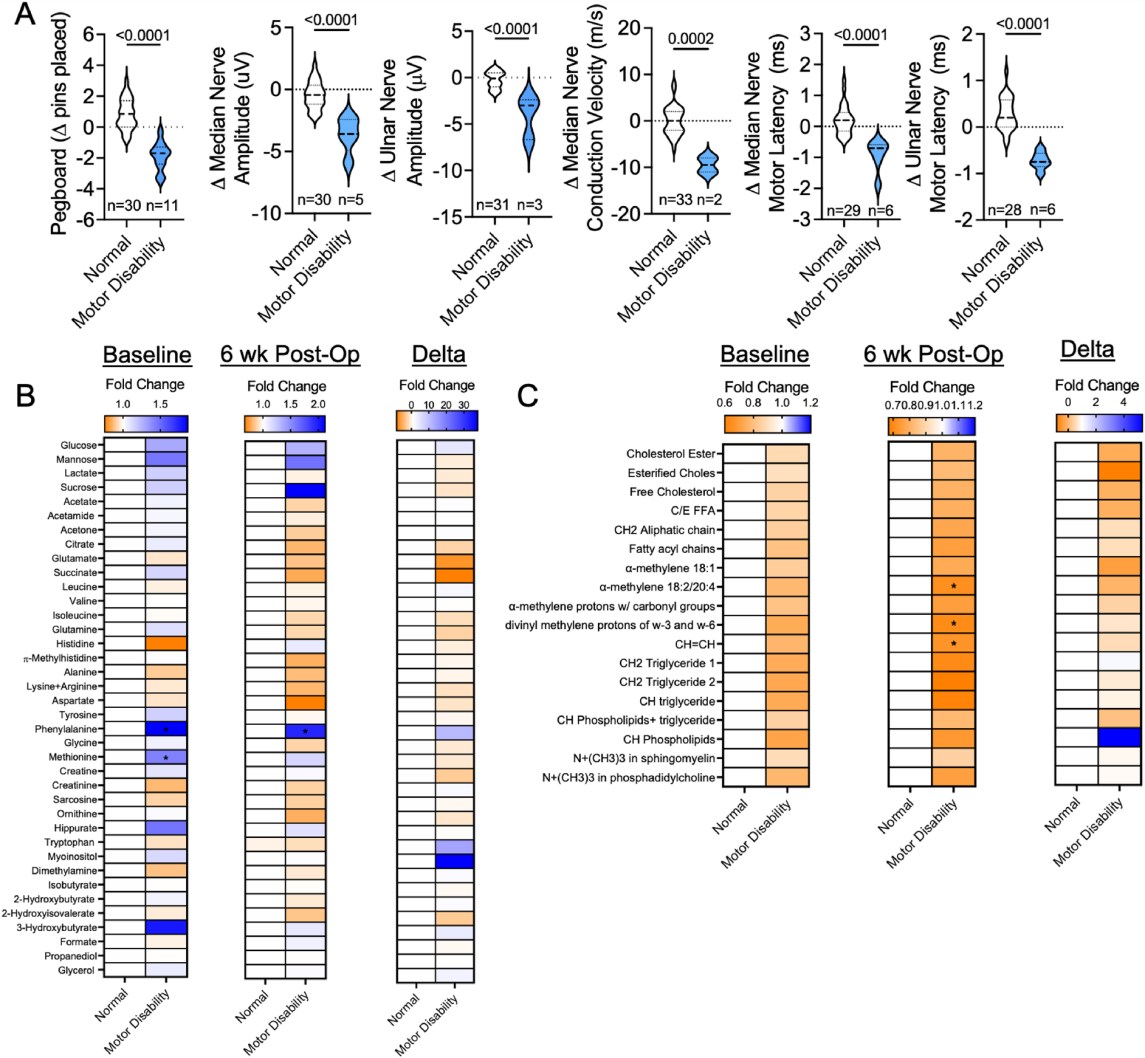
Phenylalanine accumulation is a unique characteristic of ARHD patients with motor disability. (**A**) Violin plots showing changes in the Purdue Pegboard and motor nerve conduction tests. (**B**) Heatmaps showing the fold change in aqueous phase metabolites at baseline (pre-operative), 6 weeks post-operatively, and the change for patients classified as ‘normal’ and those with ‘motor disability’. (**C**) Heatmaps showing the fold change in lipid phase metabolites at baseline (pre-operative), 6 weeks post-operatively, and the change for patients classified as ‘normal’ and those with ‘motor disability’. Comparisons between two groups were performed using an unpaired Student’s* t*-test. Metabolite comparisons were performed using an unpaired Student’s* t*-test. with FDR correction for multiple comparisons.

Decreases in dexterity and motor discoordination are also reported as components within the spectrum of ARHD following AVF creation. Accordingly, we categorized patients into those with either “normal” motor function or “motor disability” based on their performance on the Purdue Pegboard test and motor nerve conduction examination. Ten patients were classified as having motor disability by demonstrating decreased performance in the Purdue Pegboard test, as well as having alterations in either amplitude, conduction velocity, or latency of the ulnar or median motor nerves (Fig. 3A). Analysis of the baseline serum metabolites revealed that significant elevations in phenylalanine and methionine distinguished patients with motor disability from those without (Fig. 3B). Interestingly, only the elevation in phenyalanine separated patients with or without motor disability at the 6-week post-operative timepoint (Fig. 3B). Correspondingly, a change in metabolite concentration between baseline and 6 weeks post-AVF creation was not able to distinguish patients that exhibited motor disability from those without (Fig. 3B). No lipid metabolite levels at baseline were able to distinguish the two patient groups (Fig. 3C). Post-operatively, a decrease in unsaturated lipids (*P* = 0.038) and polyunsaturated lipids (omega 3/6) were associated with patients that had motor disability (Fig. 3C). There were also no changes in lipid metabolite levels when comparing pre-operative to post-operative biomechanical and neurophysiological outcomes that were able to distinguish the two groups. Please refer to Supplemental Table 1 for additional details regarding all relevant metabolites that were examined during the study for each patient.

### Altered amino acid levels distinguish ARHD patients with mild sensory disability from those without sensory alterations

**Figure 4:**
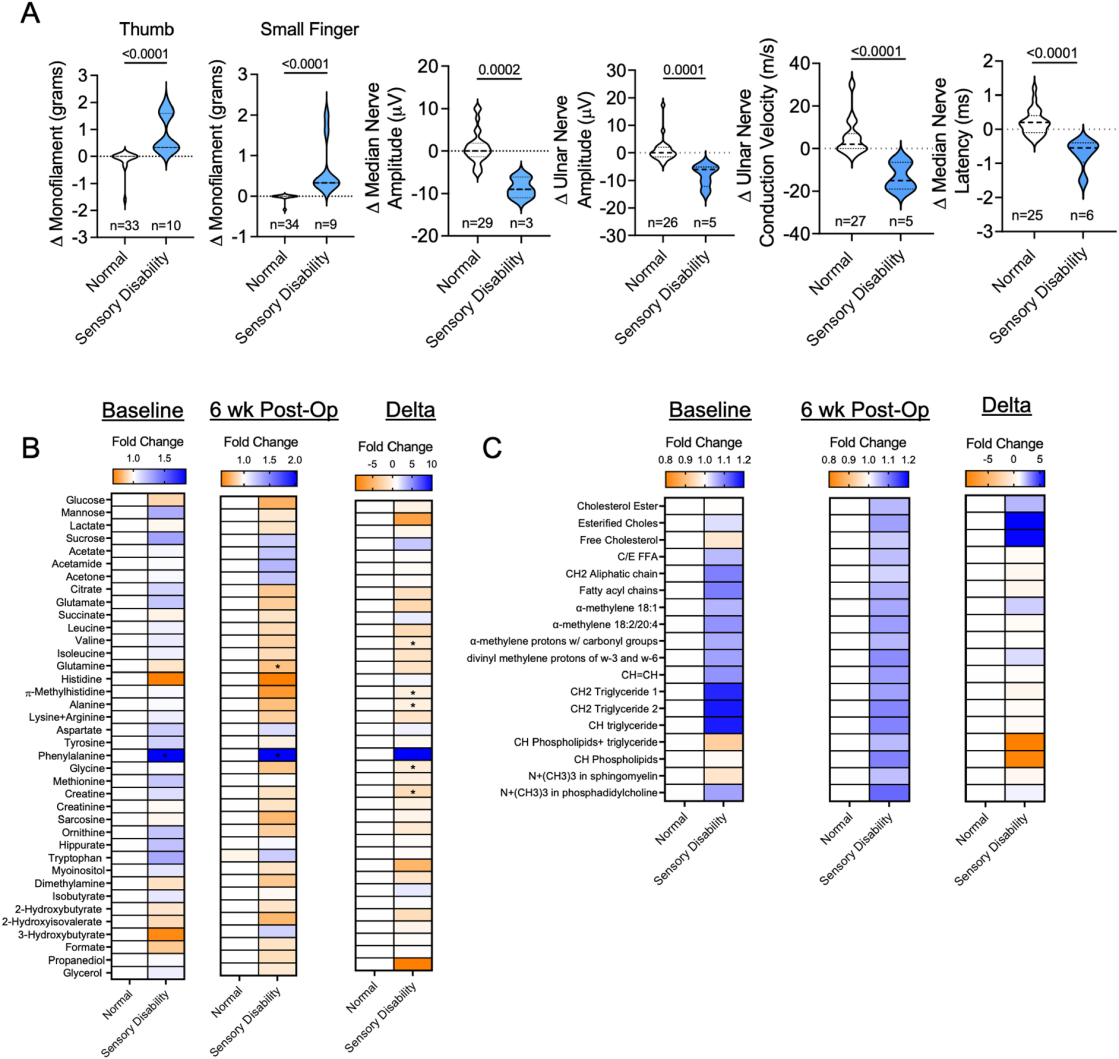
Decreased amino acid levels are found in ARHD patients with sensory disability. (**A**) Violin plots showing changes in monofilament and sensory nerve conduction tests in patients classified as having mild sensory disability and those without (normal). (**B**) Heatmaps showing the fold change in aqueous phase metabolites at baseline (pre-operative), 6 weeks post-operatively, and the change for patients classified as ‘normal’ and those with ‘sensory disability’. (**C**) Heatmaps showing the fold change in lipid phase metabolites at baseline (pre-operative), 6 weeks post-operatively, and the change for patients classified as ‘normal’ and those with ‘sensory disability’. Comparisons between two groups were performed using an unpaired Student’s* t*-test. Metabolite comparisons were performed using an unpaired Student’s* t*-test. with FDR correction for multiple comparisons.

In addition to weakness and motor dysfunction, some patients that develop ARHD experience sensory impairments including reduction in light touch or pressure and fine point discrimination distal to the AVF location. To investigate sensory deficits in this patient cohort, Semmer-Weinstein Monofilament testing and sensory nerve conduction analyses were completed. Based on these analyses, fourteen patients were characterized as displaying sensory disability based on monofilament and nerve conduction testing, although it is important to note that the level of sensory deficit was modest. Statistical comparisons of groups for thumb and small finger monofilament changes, as well as changes in median and ulnar sensory nerve amplitudes are shown in Fig. 4A. Analysis of the baseline serum metabolites revealed that a significant elevation in phenylalanine (*P* = 0.031) distinguished patients with mild sensory disability from those without (Fig. 4B). Post-operatively, increased phenylalanine levels and decrease glutamine levels separated patients with sensory disability from those without sensory impairment at the post-operative timepoint (Fig. 4B). Comparing differences post-operative and baseline metabolite levels, a decrease in valine, methylhistidine, alanine, glycine, and creatine were unique to patients that exhibited sensory disability (Fig. 4B). No lipid metabolite levels at baseline, 6 weeks post-operative, or a change in concentration between the timepoints were found to be statistically significant between the two patient groups (Fig. 4C).

### Severe hemodynamic disturbances following AVF creation are associated with a unique serum metabolome

**Figure 5:**
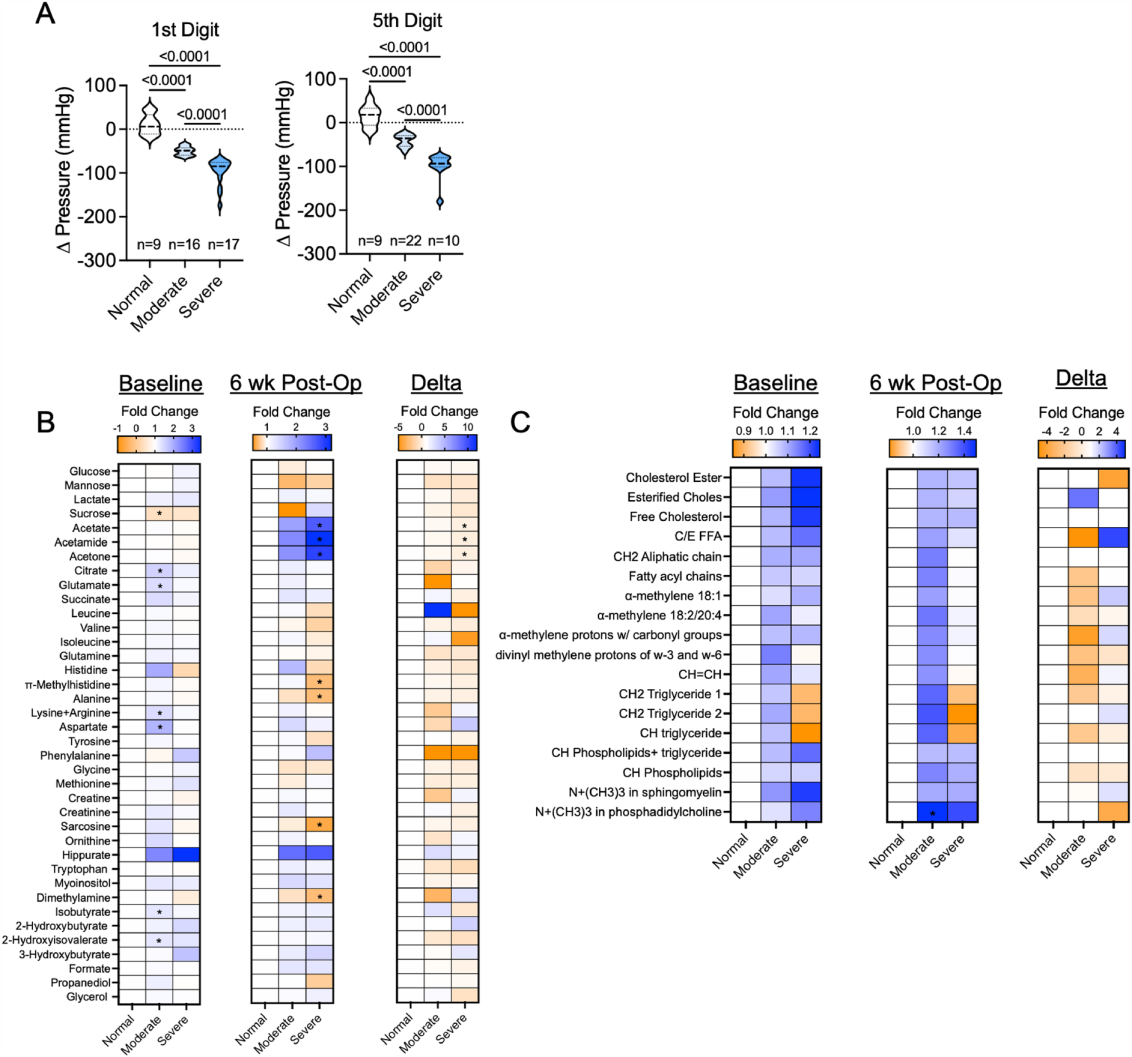
Impact of hemodynamic changes on the serum metabolome and lipidome in patients undergoing AVF surgery. (**A**) Violin plots showing changes (post-operative minus pre-operative) in the first- and fifth-digit blood pressures in patients undergoing AVF surgery. (**B**) Heatmaps showing the fold change in aqueous phase metabolites at baseline (pre-operative), 6 weeks post-operatively, and the change for patients classified as ‘normal’, ‘moderate’, and ‘severe’ hemodynamic changes. (**C**) Heatmaps showing the fold change in lipid phase metabolites at baseline (pre-operative), 6 weeks post-operatively, and the change for patients classified as ‘normal’, ‘moderate’, and ‘severe’ hemodynamic changes. Metabolite comparisons were relative to patients with normal hemodynamics using an unpaired Student’s* t*-test with FDR correction for multiple comparisons.

Creation of the AVF invariably generates some hemodynamic alterations distal to the site of anastomosis, although our previous work found that these changes correlated poorly with the degree of ARHD as measured by changes in biomechanical hand outcomes^4^. To explore whether serum metabolome changes might be associated with hemodynamic alterations with AVF placement, we segmented the patient population into those with normal, moderate, and severe hemodynamic alterations based on the average change in the first- and fifth-digit pressures measured by non-invasive vascular laboratory testing post-operatively. Eight patients in total were found to have no meaningful change in digit pressures, whereas nineteen patients had mild decreases (15–65 mmHg) in digit pressures and thirteen patients had severe decreases in digit pressures (> 70 mmHg) (Fig. 5A). Notably, a few patients had mild decreases in only a single digit pressure and thus the average pressures resulted in placement into the ‘normal’ group. Analysis of the baseline (pre-operative) serum metabolites identified several modestly elevated metabolites (citrate, glutamate, aspartate, lysine/arginine, isobutyrate, 2-hydroxyisovalerate) that were consistently unique to patients that experienced moderate hemodynamic changes (Fig. 5B). Post-operatively, patients that experienced severe hemodynamic alterations also had higher levels of acetate, acetamide, and acetone when compared to patients with normal digit pressures (Fig. 5B), although this effect was largely driven by a decrease in the concentration of these metabolites in patients with normal digit pressures. Additionally, these patients had decreased levels in methylhistidine, alanine, sarcosine, and dimethyl amine when compared to patients with normal digit pressures (Fig. 5B). Comparing within patient differences for post-operative and baseline metabolite levels, patients with severe hemodynamic changes experienced a larger relative decrease in acetate, acetamide, and acetone. Analysis of the serum lipidome revealed that the only difference detected was a significant increase in phosphatidylcholine levels post-operatively in patients with mild hemodynamic alterations (Fig. 5C).

## Discussion

This study was the first to interrogate whether serum metabolomic and/or lipidomic changes in CKD/ESKD patients undergoing AVF surgery could distinguish between subjects that develop ARHD from those who do not by 6 weeks postoperatively. Among the different domains that comprise aspects of ARHD that were investigated for this analysis, biomechanical outcomes displayed the largest change. Specifically, 19 out of the 45 patients experienced a significant decrease in grip strength 6 weeks post-AVF creation. Metrics of motor and sensory nerve function displayed smaller changes comparably. Unfortunately, the serum metabolome and lipidome were not sufficiently different between patients that experienced weakness (decrease in grip strength) and those that did not experience weakness to allow for cohort discrimination (Fig. 2). This observation indicates that a blood-based biomarker for identifying patients most at risk for developing hand weakness post-AVF surgery was not found within our dataset. Interestingly, we found a significant correlation between changes in serum tryptophan levels and the post-operative change in grip strength (*P* = 0.019), indicating that patients that developed weakness had decreases in tryptophan levels. Relevant to this observation, several well-known uremic solutes/toxins including indoles and kynurenines, which accumulate in CKD^8,10,19–23^, are derived from tryptophan catabolism. Moreover, several of the tryptophan-derived uremic solutes have been directly linked to muscular pathology including atrophy, weakness, and mitochondrial impairments^22,24–28^. Unfortunately for ESKD patients, several of these uremic solutes are protein bound and not removed effectively using contemporary clinical dialysis membranes^9^. Taken together, the findings from this analysis support the need for future studies to determine if tryptophan-derived uremic solutes have a causal role in the development of ARHD.

In contrast to clinically measured grip strength, fewer patients displayed objective indicators of motor (n = 10) or sensory (n = 14) nerve conduction impairment following AVF placement, and all of these were considered mild in nature. In fact, some patients displayed alterations in motor and sensory nerve conduction assessments, but these did not manifest clinically as changes in the Pegboard or monofilament tests. This observation may suggest the possibility that some patients exhibit sub-clinical motor/sensory deficits that could materialize clinically with longer follow-up. Regarding metabolites, patients with motor nerve disability had greater levels of phenylalanine at both timepoints when compared to patients classified as having normal motor nerve function (Fig. 3B). It is well known that the kidney plays a crucial role in the uptake of phenylalanine and its conversion to tyrosine^29,30^. Interestingly, our recent analysis of the muscle metabolome in a murine AVF model also identified that high levels of muscle phenylalanine were associated with weakness (hindlimb grip strength) and motor disability (walking speed)^16^, suggesting that serum phenylalanine may be a biomarker for these phenotypic characteristics of ARHD.

Similarly, patients that exhibited mild sensory disability also exhibited altered serum amino acid profiles when compared to those without sensory impairment (Fig. 4B). Specifically, patients with sensory disability exhibited significant decreases in valine, alanine, glycine, and methylhistidine. Altered amino acid metabolism is considered a hallmark of CKD^31^, however these alterations have yet to be mechanistically linked to the development of ARHD in CKD/ESKD patients undergoing dialysis access surgery. Interestingly, among CKD mice, similar decreases in glycine and alanine were detected in the hindlimb skeletal muscle following creation of an iliac AVF^16^. More intriguingly, a recent study reported that deficiency in serine and glycine drives diabetic peripheral neuropathy in mice^32^ which could be rescued by dietary serine supplementation. These findings suggest a potential novel and pragmatic prophylactic clinical intervention that could be tested in CKD/ESKD patients undergoing AVF placement as a means of attenuating the risk of developing ARHD postoperatively.

The present study has some limitations that warrant discussion. First, this study performed metabolomic and lipidomic analyses using NMR-based technologies, which is highly regarded for its superior accuracy, but has lower sensitivity compared with mass spectrometry. Thus, we were unable to detect many metabolites that are less concentrated (NMR limited to metabolites in the high micromolar range generally). Furthermore, the extraction process may have influenced the detectable metabolites, however all samples were processed using an identical extraction protocol. It is likely that there are more metabolite alterations post-AVF placement that could be related to the development of ARHD. Second, metabolomic and lipidomic analyses herein were performed on blood samples obtained pre- and post-operatively only at 6-weeks. Future studies may need to assess additional timepoints post-operatively which might yield more metabolite changes with stronger association to hand disability. Given the hemodynamic alterations driven by AVF creation, blood-based biomarkers may not reflect the local tissue-level metabolomic and lipidomic alterations that are occurring within the hand/arm muscle and peripheral nerve tissues. However, it is important to note the difficulties in obtaining these types of tissues from patients. Finally, PCA analysis (Fig. 1) revealed distinct grouping of several post-operative samples using aqueous phase metabolites. Further analysis suggested that acetamide, acetate, glucose, and lactate may have been strong drivers of this grouping, however future studies with larger sample sizes are needed to account for clinical, environmental, and physical characteristics which could have contributed to the observed clustering.

## Conclusions

Herein, we performed metabolomic and lipidomic analyses of blood samples obtained pre- and post-operatively from CKD/ESKD patients receiving AVF surgery. Comparisons of the pre-operative, post-operative, and temporal changes in these metabolites were made between patients that developed symptoms of ARHD and those that did not. Post-operative decreases in several amino acid metabolites including phenylalanine, glycine, and alanine were found to be unique to patients that developed signs of motor or sensory disability. A significant correlation was found between the change in tryptophan levels and the change in grip strength suggesting a possible role of tryptophan-derived uremic metabolites in post-AVF hand-associated weakness.

### Supplementary Information

Supplementary Table 1.

## Data Availability

All raw NMR data associated with this study are available in Metabolomics Workbench using the following Study ID’s: ST002418 and ST002417. Any other requests for data herein should be directed to the corresponding author, Dr. Salvatore Scali.
